# Agreement of point of care ultrasound and final clinical diagnosis in patients with acute heart failure, acute coronary syndrome, and shock: POCUS not missing the target

**DOI:** 10.1007/s11739-024-03639-y

**Published:** 2024-06-12

**Authors:** José Atilio Núñez-Ramos, Dagoberto Duarte-Misol, María Andrea Burgos Petro, Keren Jemima Sarmiento Pérez, Vanessa Paola Gutiérrez Echeverry, Sergio Velasco Malagón

**Affiliations:** 1 Health Science Division, Universidad del Norte, Barranquilla, Colombia; 2 Emergency Department, Hospital Universidad del Norte, Soledad, Colombia; 3 Department of Internal Medicine, Universidad Nacional de Colombia, Bogotá, Colombia; 4 Grupo de Interés en Ultrasonido Enfocado UN-HUN., Bogotá, Colombia; 5 Department of Internal Medicine, Clínica Nueva El Lago, Bogotá, Colombia

**Keywords:** Point-of-care ultrasound, Diagnosis, Agreement, Heart failure, Shock, Acute coronary syndrome

## Abstract

Point-of-care ultrasound (POCUS) is an important tool for clinical diagnosis and decision-making in critical and non-critical scenarios. Dyspnea, chest pain, and shock are conditions susceptible to evaluation with ultrasound considering diagnostic accuracy and clinical impact already proven. There is scarce evidence in diagnosis agreement using ultrasound as an extension of physical examination. We aimed to evaluate ED patients in whom POCUS was performed, to analyze agreement between clinical initial diagnosis using ultrasound images and final diagnosis. Furthermore, we analyze failed diagnosis, inconclusive POCUS exams, and discuss details. A cross-sectional analytical study was conducted on adults who visited the emergency department with any of these three chief complaints: dyspnea, chest pain, and shock. All were evaluated with ultrasound at admission. Agreement between initial diagnosis using POCUS and final definite diagnosis was calculated. Failed diagnosis and inconclusive exams were analyzed. A total of 209 patients were analyzed. Populations: mostly males, mean age 64 years old, hypertensive. Agreement on patients with dyspnea and suspicion of acute decompensated heart failure was 0.98; agreement on chest pain suspicion of non-ST acute coronary syndrome was 0.96; agreement on type of shock was 0.90. Among the population, 12 patients had an inconclusive POCUS exam, and 16 patients had a failed diagnosis. The use of POCUS in the emergency department shows almost perfect agreement when compared with the final diagnosis in individuals experiencing acutely decompensated heart failure, acute coronary syndrome, and shock. Prospective studies are needed to evaluate the impact of this tool on mortality and prognosis when there are diagnostic errors.

## Introduction

Point-of-care ultrasound (POCUS) is an important tool for clinical diagnosis and decision-making in critical and non-critical scenarios. To this moment, ultrasound (US) has been used for more than 20 years in intensive care units (ICU) and emergency departments (ED) around the world. The impact on diagnosis and clinical outcomes is evident. Several accuracy studies have proved its diagnostic value over physical examination alone, and chest X-ray [1]. Clinical impact has been evaluated through cohort studies and clinical trials, demonstrating better outcomes in patients who received POCUS evaluation [2–5].

Ultrasound at the bedside has been used for critical or emergent conditions like dyspnea, chest pain, and shock, among others. Regarding shock, POCUS has proved to be of great value in a recently published accuracy study reporting a sensitivity and specificity of 98 and 96% when POCUS is used alongside history and physical examination [6]. In patients with shortness of breath, diagnosing dyspnea using lung ultrasound is better than using clinical examination and/or chest X-ray [1]. Specifically, recent guidelines recommend lung POCUS as an adjunctive diagnostic test for acutely decompensated heart failure (ADHF) [7]. Chest pain is a complex clinical presentation in the ED, and ultrasound has shown the ability to narrow the differential diagnosis in critical scenarios. Aortic dissection and acute coronary syndrome (ACS) are two of the main diagnoses in which US is helpful [8].

Since the introduction of point-of-care ultrasonography, many studies have evaluated the agreement between POCUS-specific findings compared to comprehensive ultrasonography, which is the ultrasound evaluation performed by radiologists and/or cardiologists as experts in the field. B lines, pleural effusion, dilated heart cavities, valvulopathies, and many other specific alterations have been analyzed [9–12]. Most studies show good agreement between novice operator-performed ultrasound and experienced operator-performed ultrasound. Reported evidence includes findings in echocardiography with good agreement [9] and others with a not-so-good agreement [10, 11]. Lung ultrasound is valuable and reproducible [8]. In a population of patients with shock, studies have conflicting results [12] considering a heterogeneous population and different levels of training.

There is scarce evidence comparing initial clinical diagnosis using ultrasound as an extension of physical examination and final diagnosis in clinical practice, however, evidence on diagnostic accuracy and guidelines has been published [13]. Baid et al. published a paper using POCUS as an initial diagnostic tool in the ED with good concordance with final diagnosis [14]. Buhumaid showed good differential narrowing using US but did not analyze concordance with the final diagnosis [8]. Knowing diagnostic agreement could lead to a more confident diagnosis at the emergency department and possibly to more specific and rapid treatment initiation.

We aimed to evaluate ED patients in whom POCUS was performed and analyze the agreement between clinical initial diagnosis using ultrasound images and final diagnosis at discharge in specific groups of patients. Furthermore, we analyze failed diagnosis, and inconclusive POCUS exams and discuss details.

## Methods

This is an analytical cross-sectional study of emergency department patients consulting for dyspnea, chest pain, and undifferentiated shock state; these patients were suspected of acute decompensated heart failure, acute coronary syndrome, and specific types of shock respectively. It was carried out in a tertiary-level university-based hospital in Barranquilla, Colombia. The Ethics Committee and Institutional Research Board approved the protocol (Act 281, November 24th, 2022).

### Population

The study was conducted between 2019 and 2022. Patients eligible were adults who visited the emergency department with any of these three chief conditions: acute dyspnea suspicion of acute heart failure; acute chest pain suspicion of acute coronary syndrome; shock state defined as clinical hypoperfusion (altered mental status, long capillary refill time) and/or hypotension (Mean arterial pressure less than 65 mmHg). Inclusion criteria were a POCUS evaluation performed at admission, US findings reported in clinical records, reported initial diagnosis using POCUS alongside usual clinical evaluation, reported discharge (final) diagnosis considering evolution, laboratory parameters, and images. Exclusion criteria were death during the first 24 h of admission, ST-segment elevation ACS, not definitive diagnosis at discharge, in-hospital transfer to another healthcare facility, and not clear diagnosis at entrance.

### Ultrasound and clinical evaluation

Patients in the ED with any of the three main complaints underwent a triage classification. Priority was given according to triage. The first medical contact was an emergency physician who took a directed clinical history and physical examination. Patients with acute shortness of breath (defined as dyspnea that began in the last 4 weeks) and a history consistent with congestion (edemas, jugular distension, S3 gallop, hepatojugular reflux) were considered to have acute decompensated heart failure. Patients consulting with cardiac chest pain considered acute coronary syndrome were approached accordingly. Shock patients were treated in a resuscitation room and received intravenous fluids if considered hypovolemic, inotropes if the initial diagnosis was cardiogenic shock, and antibiotics plus cultures if deemed septic shock.

After first medical contact, focused clinical history was taken by the attending internal medicine physician. A thorough physical examination was performed, including Point-of-Care Ultrasound as an extension of the physical evaluation to assure an initial diagnosis. Every patient had an initial diagnosis using clinical history, physical examination, and ultrasound evaluation. After clinical evaluation, a POCUS exam and a decision made, initial treatment was ordered.

In patients with a suspicion of ADHF, diagnostic aids, and interventions included oxygen, intravenous furosemide, chest X-ray, lung ultrasound, cardiac ultrasound, electrocardiogram, natriuretic peptides, etc. Diagnostic aids and interventions for patients with a suspicion of ACS included dual antiplatelet inhibition, sublingual or intravenous nitrates, parenteral anticoagulation, atorvastatin, comprehensive echocardiogram, and troponin assay.

POCUS evaluation was performed using a multiprobe ultrasound machine (Sonoscape Corp. Model S2. Guangdong, China. 2016-3) by the attending internal medicine physician trained in POCUS. Dyspneic patients assessment included BLUE protocol [15] using a linear transducer with a frequency of 5–10 MHz and a phased array with a frequency of 2–5 MHz. B-lines, pleural effusion, and lung consolidation were specific findings at US evaluation. Findings were integrated with clinical symptoms to consider a specific initial diagnosis.

Patients with acute chest pain were evaluated with a focused cardiac ultrasound using a phased array probe with a frequency of 2–5 MHz. In patients with a suspicion of acute coronary syndrome, POCUS analyzed at least three of the four main cardiac views (parasternal long and short axis, apical, and subxiphoid). Eyeball systolic ventricular function, pericardial effusion, and wall motion abnormalities were considered. RUSH protocol [16] was followed in shock patients to rule-in or rule-out specific etiologies such as obstructive causes, cardiogenic shock, vasodilated shock or hypovolemic shock.

Patients were treated according to the initial diagnosis and always after the POCUS exam. Medications, images, and laboratory tests were ordered considering clinical context and up-to-date clinical guidelines. No further ultrasound evaluation was performed during hospitalization.

The internal medicine specialist from the ED did not have any other interaction with the patient or clinical staff in the ICU and general ward. Specialists from these clinical areas were aware of the POCUS initial evaluation.

### Final diagnosis

The final diagnosis was given by the attending physician at discharge in the general ward. This internal medicine specialist oversaw every patient included in the study from in-ward admission until discharge. This diagnosis considered initial evaluation, all laboratory diagnostic work-up, images, and clinical evolution to declare a final and definite diagnosis.

### Data and statistical analysis

All the information was taken from clinical records. Every variable was drawn from initial clinical history, ultrasound evaluation at admission, and final diagnosis on discharge day.

According to published methods for sample size estimation [17], considering a disagreement probability of 20%, a Cohen’s Kappa of 0.85, and an alfa value of 0.05, the population should be of 192 patients. Moreover, we report sensitivities, specificities, predictive values, and likelihood ratios in each group of patients.

Categorical variables are expressed in absolute and relative frequencies. Continuous variables are expressed in mean and standard deviation, median, and interquartile ranges. The initial diagnosis was compared for agreement with the final diagnosis using Cohen’s kappa statistic in patients with heart failure, chest pain, and shock. A two-sided *p* value < 0.05 was considered to indicate statistical significance. All statistical analyses were conducted using SPSS version 25.

## Results

**Fig. 1:**
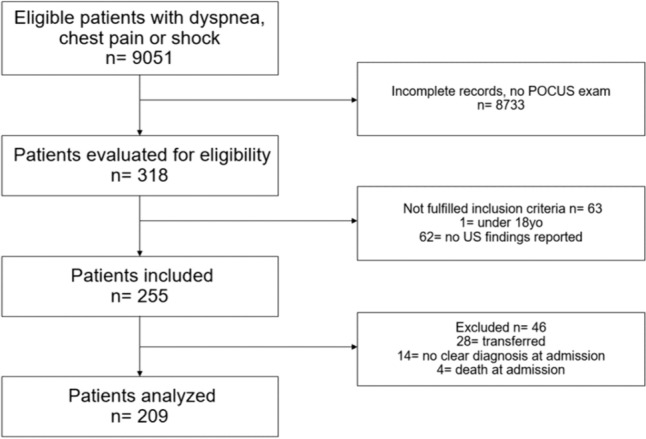
Please provide figure caption

There were 9051 patients presenting to the ED with dyspnea, shock, or chest pain in the specified period. Out of the total population, 318 (3.5%) patients received a POCUS evaluation at admission. Patients not fulfilling inclusion criteria were 63 (0.7%) and 255 (2.8%) were included. There were 46 (0.5%) patients excluded. Finally, 209 patients were analyzed (Fig. 1).

The population was predominantly male (56%), mean age of 64 years old, with a background of hypertension (60%) and type 2 diabetes (23%). The main reason to visit the ED was dyspnea (56.9%), chest pain (29.2%), and shock (13.9%). The most frequent diagnosis at the entrance was acute decompensated heart failure (38.3%) followed by acute coronary syndrome (17.2%) (Table 1).

**Table 1:** Population characteristics

Variables	All *n* = 209
Demographic and clinical	*n* (%)
Age years (Mean, SD)	64 (17)
Male sex	118 (56,5)
Hypertension	126 (60)
Type 2 diabetes	49 (23)
Heart failure	41 (20)
Coronary heart disease	51 (24)
POCUS indication	*n* (%)
Dyspnea	119 (56,9)
Chest pain	61 (29,2)
Shock	29 (13,9)
Imaging studies	*n* (%)
Chest X-ray	174 (83,3)
Echocardiogram	153 (73,2)
Chest CT scan	78 (37,3)
POCUS findings	*n* (%)
Interstitial syndrome	158 (75,4)
Pleural effusion	72 (34,5)
Lung consolidation/atelectasis	84 (40,1)
Left ventricle dysfunction	109 (52,3)
Wall motion abnormalities	97 (46,3)
Pericardial effusion	24 (11,5)
Non-conclusive	12 (5,7)

A total of 153 patients (73.2%) received a comprehensive echocardiographic evaluation by a cardiologist. Regarding other imaging studies, 174 (83.2%) patients had a chest X-ray at the ED entrance and 78 (37.3%) had a CT scan (Table 1).

All patients were evaluated with POCUS at the ED entrance. Cardiac findings include eyeball ventricular systolic dysfunction (left ventricle ejection fraction < 50%) in 52.3% of the patients, 46.3% of evaluations had wall motion abnormalities, 16.3% of cardiac evaluations revealed dilated right ventricle and only 11.5% of patients had pericardial effusion. Regarding lung ultrasound, 75.4% of exams were positive for interstitial syndrome according to BLUE protocol; 34.5% reported pleural effusion, 25.3% encountered lung consolidation and 14.8% showed atelectasis. Out of the 209 POCUS evaluations, 12 patients (5.7%) had a non-conclusive exam. The number of missed diagnosis or non-concordant diagnosis was 16 (13%) (Table 1).

### Acute heart failure

Analyzing this group of patients, we found a total of 78 patients with a final diagnosis consistent with acute decompensated heart failure out of 119 patients consulting with dyspnea. POCUS correctly identified all ADHF patients. Two patients were inadequately classified as acute decompensation, the final diagnoses for these two patients were pneumonia and urinary tract infection. Patients were discharged with no complications.

Overall concordance between initial diagnosis and final diagnosis in patients consulting with dyspnea suspicion for ADHF diagnosis resulted in a Cohen’s Kappa of 0.98 (Table 2). Sensitivity 100%, specificity 98%, positive predictive value 97%, negative predictive value 100%, positive likelihood ratio 65, negative likelihood ratio < 0.001, diagnostic accuracy 99 (Table 3).

**Table 2:** POCUS findings and agreement in each group

Dyspnea *n* = 119	*n* (%)
Interstitial syndrome (BLUE protocol)	112 (94,1)
Pleural effusion	40 (33,6)
Pericardial effusion	16 (13,4)
Left ventricle dysfunction	70 (58,8)
Regional wall motion abnormalities	42 (35,3)
Consolidation/atelectasis	22 (18,5)
Final heart failure diagnosis	78 (65,5)
Agreement (Cohen’s Kappa)	0,98
Chest pain *n* = 61	*n* (%)
Interstitial syndrome (BLUE protocol)	18 (29,5)
Pleural effusion	5 (8,2)
Pericardial effusion	4 (6,6)
Left ventricle dysfunction	27 (44,3)
Regional wall motion abnormalities	38 (62,3)
Consolidation/atelectasis	2 (3,3)
Final non-ST acute coronary syndrome	58 (95)
Agreement (Cohen’s Kappa)	0,96
Shock *n* = 29	*n* (%)
Interstitial syndrome (BLUE protocol)	5 (17)
Pleural effusion	4 (14)
Pericardial effusion	1 (3)
Left ventricle dysfunction	5 (17)
Regional wall motion abnormalities	1 (3)
Consolidation/atelectasis	5 (17)
Final diagnosis of shock	25 (86)
Agreement (Cohen’s Kappa)	0.90

**Table 3:** Diagnostic accuracy for each clinical entity

	Heart failure	Non-ST ACS	Shock (CI 95%)
Sensitivity	100% (95–100)	91% (77–98)	84 (65–95)
Specificity	98 (94–99)	100 (97–100)	100 (98–100)
Positive predictive value	97 (90–99)	100 (89–100)	100 (84–100)
Negative predictive value	100 (97–100)	98 (95–99)	98 (95–99)
Positive likelihood ratio	65 (16–259)	>99	>99
Negative likelihood ratio	<0.001	0.08 (0.03–0.25)	0.15 (0.06–0.38)
Diagnostic accuracy	99 (96–99)	98 (96–99)	98 (95–99)

*Values are expressed in percentage (CI 95%)

### Acute coronary syndrome

In this group of patients, a total of 58 patients were finally classified as non-ST acute coronary syndrome out of 61. All were correctly diagnosed using clinical history, physical exam, and ultrasound. There were three patients initially classified as having ACS who finally were diagnosed with hypertensive angina (two of them) and one with osteochondritis.

We found only 28 patients with regional wall motion abnormalities (RWMA) in comprehensive echocardiography and 23 of those patients were correctly identified by POCUS. Five patients were identified with RWMA by comprehensive echocardiography, which was not identified during the POCUS examination, and five patients with RWMA were identified by POCUS but not comprehensive echocardiography. Cohen´s Kappa in this subgroup of patients was 0.40. Overall concordance between initial diagnosis and final diagnosis in patients consulting with acute chest pain with non-ST ACS diagnosis resulted in a Cohen´s Kappa of 0.96 (Table 2). Sensitivity 91%, specificity 100%, positive predictive value 100%, negative predictive value 98%, positive likelihood ratio > 99, negative likelihood ratio 0.08, diagnostic accuracy 98 (Table 3).

### Shock

In this group, we found 26 patients with a final diagnosis of shock. Regarding the type of shock, 17 patients had septic shock, 5 were diagnosed with cardiogenic shock, 3 with shock and dilated right ventricle (supposedly obstructive), and 1 with hypovolemic shock.

Ultrasound and clinical evaluation correctly identified the type of shock in 22 patients. Four patients were initially misclassified. Two patients had an initial non-conclusive ultrasound exam. No specific findings were seen and alongside history were classified as non-conclusive. Finally, these two patients were diagnosed with septic shock. One patient had a dilated right ventricle and was classified as obstructive shock, but at discharge was given a diagnosis of septic shock. The fourth patient was identified as having COPD exacerbation at ED admission, but at the end of hospitalization was classified as septic shock.

Overall concordance between the initial diagnosis of the type of shock resulted in a Cohen’s Kappa of 0.90 (Table 2). Sensitivity 84%, specificity 100%, positive predictive value 100%, negative predictive value 98%, positive likelihood ratio > 99, negative likelihood ratio < 0.15, diagnostic accuracy 98 (Table 3).

### Non-conclusive POCUS exams

Out of 209 ultrasound evaluations 12 were reported as non-conclusive. This report was given considering a normal ultrasound evaluation during which no lung abnormality was found, cardiac ultrasound was normal with a good quality image of normal systolic function. This report was applied when there was not a specific finding that could explain the patient’s symptoms or any abnormality that may serve as a diagnosis support.

There were 6 patients with pneumonia not identified at initial POCUS evaluation, 2 patients with septic shock, and one patient with each of these diagnoses: non-cardiac chest pain, hypertensive angina, obstructive sleep apnea, and soft-tissue infection. (Table 2).

## Discussion

In this real-life study, an almost perfect concordance was observed between the point-of-care ultrasound POCUS-guided diagnosis conducted in the emergency department and the final diagnosis given at discharge for patients presenting with acute dyspnea suspicion of ADHF, acute chest pain suspicion of ACS or shock. This finding highlights the utility of POCUS as an effective tool for evaluating patients in these clinical scenarios and the high confidence that should be placed on a multiorgan ultrasound evaluation alongside a clinical exam.

In every case, the categorization of individuals with acute heart failure was accurate. Gallard et al. [18] conducted a study involving a cohort of 130 patients to assess the diagnostic accuracy of heart failure. Their findings indicated a diagnostic accuracy of 90% which aligns with our research. Baid et al. [14] found a sensitivity of 77% in diagnosing left ventricular systolic dysfunction. Previous research has indicated that emergency physicians have achieved a detection rate of 82%. The agreement observed in our study, using multimodal Point-of-care ultrasound was 0.98. This result contrasts with the findings reported by Baid et al., who reported agreement values of 0.59 for systolic dysfunction and 0.83 for pulmonary edema.

Our agreement value could be explained by the different approach in our research. The clinical picture plus physical exam and ultrasound evaluation, these three aspects as a global clinical-ultrasound approach that combines clinical reasoning using POCUS may optimize clinical conclusions. Conducting a complete clinical history, identifying clinical signs of congestion, and confirming these findings with a well-executed ultrasound evaluation form the basis of a well-founded diagnosis.

In the ACS group, adequate classification was performed on 61 patients, yielding a kappa agreement value of 0.96. Prior studies have shown variability in the accuracy of POCUS diagnosing acute coronary syndrome. Zanobetti et al. established a concordance of 0.70 between the initial diagnosis of acute coronary syndrome and the final diagnosis in emergency department patients presenting with dyspnea [19]. This study evaluated POCUS diagnoses versus ED diagnoses, but our study evaluated a different scenario: initial diagnosis using POCUS versus final diagnosis. Our work showed better performance of a clinical approximation to patients including cardiac ultrasound and integrating findings to clinical diagnosis. We are aware that focused cardiac ultrasound neither confirms nor discards a patient having ACS, but we believe that ruling out emergent entities such as aortic dissection or cardiac tamponade brings value to the ultrasound evaluation and permits a more confident ACS diagnosis.

Regarding regional wall motion abnormalities (RWMA), accuracy was not good. Croft et al. evaluated the diagnostic potential of RWMA in individuals with ST-elevation myocardial infarction in the WAMAMI study [20] reporting a Kappa 0.79 after specific training of emergency medicine residents. Again, not a clinical diagnosis but a specific ultrasound finding. Another study conducted by Saglam [21] in patients with suspected non-ST myocardial infarction reported a good agreement of 0.84 after a 3-h training. Our data show a low agreement (kappa 0.4), thus, reflecting the high variability of this specific ultrasound finding and the need to not rely only on RWMA to diagnose or treat patients with chest pain.

Analyzing the shock population, we found good agreement between the initial POCUS-guided diagnosis and final diagnosis. A recent systematic review conducted by Yoshida et al. [6] highlights the diagnostic efficacy of point-of-care ultrasound (POCUS) in this set of patients, showing over 95% diagnostic accuracy. We correctly identified the type of shock in 84.6% of our patients. In the septic shock group, we had two patients with inconclusive findings at admission. Septic shock is an entity with no specific ultrasound finding. Clinical history and reasoning are crucial to correct diagnosis. According to our results, a clinician should always be aware of septic shock and interpret the whole clinical picture, including POCUS, to give a shock-type diagnosis.

Previously, Javali et al. [22] demonstrated that in cases of undifferentiated shock in the emergency department, the diagnostic accuracy for identifying the type of shock increased from 45 to 89% with the addition of point-of-care ultrasound. A kappa value of 0.89 was reported and is consistent with similar findings by Vaidya et al. [23] and our data. The findings of these studies depict a scenario akin to the one outlined in our study, where clinical data was integrated with POCUS information.

We had 12 patients with a non-conclusive POCUS at admission, explaining most patients with wrong diagnosis. Analyzing these patients, we found 6 with pneumonia not diagnosed adequately. We explain these findings considering the limited sensitivity of lung ultrasound in certain scenarios. It is required that lung consolidation involves pleura and to be in an area susceptible to visualization (BLUE points), this could be a limitation for a given diagnosis. This highlights the need for an extensive lung ultrasound evaluation if BLUE points are non-conclusive, and the clinical picture is consistent with a lower respiratory tract infection. There should be more research on missed diagnoses with POCUS and its clinical impact on prognosis and mortality to comprehensively evaluate ultrasound errors.

### Limitations and advantages

This study was conducted at a single center within a university hospital, involving a specific population, which may limit the generalizability of the findings. Although we included a varied population which represents a usual ED population, caution should be taken when applying the results. Similarly, using images captured by a single provider may limit the application of our study, it reduces the potential for differences in interpretation among observers.

Considering global population consulting to our ED (*n* = 9051), we conducted POCUS evaluations only in 318 patients. This reflects the limitations of the US in an emergency department with low training rates among facultative. Moreover, the availability of ultrasound equipment could be a limitation for scanning all patients. We believe this limits the interpretation of our results and poses biases in the analysis, biases that were considered and controlled with exclusions when appropriate.

This study was conducted in an emergency department in which internal medicine is the main clinical specialty evaluating patients with dyspnea, chest pain, and shock. This may not reflect the generalized situation in other countries and cities in the world. However, we believe that clinical approaches may be very similar in these cases considering the clinical background of emergency medicine and internal medicine; moreover, using Point-of-Care Ultrasound gives the clinician an objective evaluation of the patient. Specific ultrasound findings are reliable and have proven to diagnose better than clinical exam, as we have stated and discussed previously.

The study’s strengths lie in its real-life depiction of typical conditions in the emergency department, where point-of-care ultrasound (POCUS) information is integrated into the diagnostic process. This integration enables real-time decision-making for patients with potentially life-threatening pathologies. We had a comorbid population, consulting to the ED with the most common symptoms in clinical practice, evaluated with ultrasound, and diagnosed with clear parameters at discharge. All patients had POCUS and gold standard clinical diagnosis. Statistical analyses were performed following adequate procedures and sample size was achieved giving enough power to our study.

Finally, we consider POCUS should be at the center of clinical evaluation in every emergency department given previous data and our results. Nonetheless, we send a caution message to always integrate ultrasound with clinical evaluation. The results of this study demonstrate a strong correlation with definitive diagnosis, emphasizing the importance of integrating these findings into the assessment of patients with potentially life-threatening conditions.

## Conclusion

In this study conducted in the ED, we found an almost perfect agreement when compared with the final diagnosis in individuals experiencing acutely decompensated heart failure, non-ST acute coronary syndrome, and shock. This reinforces the need for regular implementation of POCUS in clinical practice always integrating it into the clinical picture. Prospective studies are essential to evaluate the impact of this tool on mortality and prognosis when there are diagnostic errors.

## Conflict of interest

The authors have no conflict of interest or financial funding to disclose.

## Data Availability

The data that support the findings of this study are available on request from the corresponding author, Núñez-Ramos JA. The data are not publicly available due to information that could compromise the privacy of research participants.
